# Interictal Functional Connectivity of Human Epileptic Networks Assessed by Intracerebral EEG and BOLD Signal Fluctuations

**DOI:** 10.1371/journal.pone.0020071

**Published:** 2011-05-19

**Authors:** Gaelle Bettus, Jean-Philippe Ranjeva, Fabrice Wendling, Christian G. Bénar, Sylviane Confort-Gouny, Jean Régis, Patrick Chauvel, Patrick J. Cozzone, Louis Lemieux, Fabrice Bartolomei, Maxime Guye

**Affiliations:** 1 CNRS (Centre National de la Recherche Scientifique), UMR 6612, CRMBM (Centre de Résonance Magnétique Biologique et Médicale), Marseille, France; 2 INSERM (Institut National de la Santé et de la Recherche Médicale), U751, Laboratoire de Neurophysiologie et Neuropsychologie, Marseille, France; 3 Université de la Méditerranée Aix-Marseille II, Faculté de Médecine, Marseille, France; 4 Pôle d'imagerie Médicale, Assistance Publique – Hôpitaux de Marseille, Marseille, France; 5 Pôle de Neurosciences Cliniques, Assistance Publique – Hôpitaux de Marseille, Marseille, France; 6 INSERM (Institut National de la Santé et de la Recherche Médicale), U642, Rennes, France; 7 UCL (University College London), Department of Clinical and Experimental Epilepsy, UCL Institute of Neurology, London, United Kingdom; 8 MRI Unit, National Society for Epilepsy, Buckinghamshire, United Kingdom; Consejo Superior de Investigaciones Cientificas - Instituto Cajal, Spain

## Abstract

In this study, we aimed to demonstrate whether spontaneous fluctuations in the blood oxygen level dependent (BOLD) signal derived from resting state functional magnetic resonance imaging (fMRI) reflect spontaneous neuronal activity in pathological brain regions as well as in regions spared by epileptiform discharges. This is a crucial issue as coherent fluctuations of fMRI signals between remote brain areas are now widely used to define functional connectivity in physiology and in pathophysiology. We quantified functional connectivity using non-linear measures of cross-correlation between signals obtained from intracerebral EEG (iEEG) and resting-state functional MRI (fMRI) in 5 patients suffering from intractable temporal lobe epilepsy (TLE). Functional connectivity was quantified with both modalities in areas exhibiting different electrophysiological states (epileptic and non affected regions) during the interictal period. Functional connectivity as measured from the iEEG signal was higher in regions affected by electrical epileptiform abnormalities relative to non-affected areas, whereas an opposite pattern was found for functional connectivity measured from the BOLD signal. Significant negative correlations were found between the functional connectivities of iEEG and BOLD signal when considering all pairs of signals (theta, alpha, beta and broadband) and when considering pairs of signals in regions spared by epileptiform discharges (in broadband signal). This suggests differential effects of epileptic phenomena on electrophysiological and hemodynamic signals and/or an alteration of the neurovascular coupling secondary to pathological plasticity in TLE even in regions spared by epileptiform discharges. In addition, indices of directionality calculated from both modalities were consistent showing that the epileptogenic regions exert a significant influence onto the non epileptic areas during the interictal period. This study shows that functional connectivity measured by iEEG and BOLD signals give complementary but sometimes inconsistent information in TLE.

## Introduction

It has been largely demonstrated that a coherent slow fluctuation in blood-oxygen-level-dependent (BOLD) signals at rest is an accurate signature of functional connectivity between remote brain areas [1]–[3]. However despite the dramatic increase of the number of resting-state fMRI studies over the past 5 years, only a few have investigated the neural basis of such spontaneous fluctuations in fMRI signals [4], [5]. Indeed, using simultaneous BOLD and intracortical electrophysiological signal recordings of visual cortices of monkeys, Shmuel & Leopold were the first to show a correlation between slow fluctuations in BOLD signals and the underlying local neuronal activity in healthy animals [4]. In humans, He and colleagues have demonstrated a correlation structure between spontaneous BOLD fluctuations and slow cortical potentials (<4 Hz) recorded by electrocorticography (ECoG) in 5 patients during presurgical evaluation of drug-resistant partial epilepsies [5]. The authors studied specifically regions spared by electrical abnormalities in order to be in the best conditions to reproduce the physiological state. Therefore, to date, no data are available concerning the electrophysiological correlates of BOLD signal fluctuations in structures exhibiting epileptiform discharges. However, this issue is of crucial interest not only to test the clinical relevance of resting-state fMRI in the presurgical assessment of intractable epilepsies but also to better understand the functional alterations associated with these pathologies. Thus, resting-state fMRI studies have shown altered functional connectivity in language, perceptual, attention and so-called default mode networks as well as in epileptogenic networks in patients presenting with temporal lobe epilepsy (TLE) [6]–[12]. In previous studies we demonstrated decreased functional connectivity as measured by resting-state fMRI in epileptic regions, contrasting with a global increase of functional connectivity as measured by intracerebral EEG (iEEG) recording using stereo-electroencephalography (SEEG) [10], [11], [13].

Thus, in this study, we aimed to investigate the relationship between BOLD and iEEG signals by comparing the functional connectivity calculated based on the two types of data in epileptic areas and regions spared by epileptiform activities during the interictal state in 5 patients with TLE, the commonest form of partial epilepsies. We measured non-linear cross-correlations between signals obtained from the brain regions: i) involved in the generation of seizures and interictal spiking (defined as epileptogenic zone/primary irritative zone, EZ/IZ1); ii) involved only in the generation of interictal spiking (defined as secondary irritative zone, IZ2); and iii) unaffected by seizure initiation or interictal spiking (defined as non irritative zone, NIZ). We chose to use the same non-linear correlation measure for both modalities, namely h^2^, based on its relevance in electrophysiological measurements especially in epileptic patients [14].

## Results

### Classification of the regions of interest according to the epileptic process

**Figure 1:**
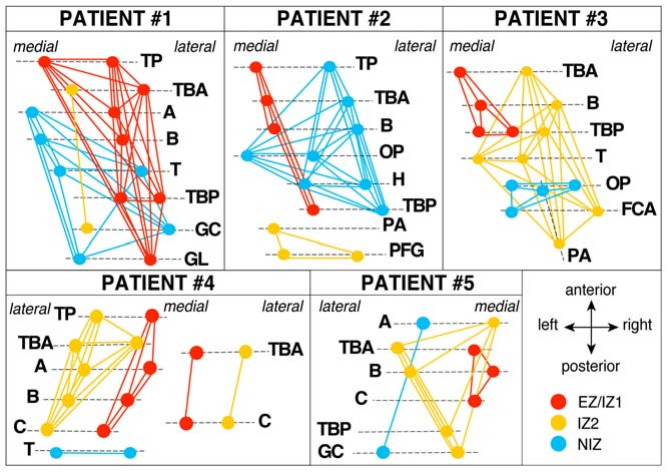
Interactions between regions of interest recorded from depth electrodes. Red circles: ROIs are in the epileptogenic/primary irritative zone (EZ/IZ1); Orange circles: ROIs are in secondary irritative zone (IZ2); Blue circles: ROIs are in non irritative zone (NIZ). Lines correspond to the links for which connectivity was evaluated. Electrodes names correspond to each brain structure recorded by the electrode. TP: temporal pole; TBA: entorhinal cortex; A: amygdala; B: anterior hippocampus; T: thalamus; TBP: fusiform gyrus, GC: posterior cingulate gyrus; GL: lingual gyrus; OP: insula; H: Heschl gyrus; PA: precuneus; PFG: posterior cingulate gyrus; FCA: anterior calcarine fissure; C: posterior hippocampus.

We identified regions of interest (ROI) for each patient representative of EZ/IZ1 (epileptogenic zone/primary irritative zone), IZ2 (secondary irritative zone) or NIZ (non irritative zone) based on both ictal and interictal iEEG data and the position of the labeled iEEG electrode contacts. We thus identified 16 regions of interest (ROIs) in Patient #1 (8 right mesial-temporal areas and 8 right lateral-temporal areas), 15 ROIs for Patient #2 (8 right mesial-temporal areas and 7 right lateral-temporal areas), 15 ROIs for Patient #3 (7 right mesial-temporal areas, and 7 right lateral-temporal areas, and 1 in the right fusiform gyrus), 16 ROIs for Patient #4 (6 electrodes in left side lobe and 2 in right side lobe; we selected 8 mesial ROIs, 8 lateral ROIs) and 10 ROIs for Patient #5 (6 left mesial-temporal areas and 4 left lateral-temporal areas). See details in Table S1. At the group level, this resulted in 50 ROIs pair-wise interactions within EZ/IZ1, 51 within IZ2 and 50 within NIZ (“within-zone” study) (Fig. 1) and 99 interactions between EZ/IZ1 and IZ2 ROIs, 110 between EZ/IZ1 and NIZ ROIs and 87 between IZ2 and NIZ ROIs (“inter-zone” study).

**Figure 2:**
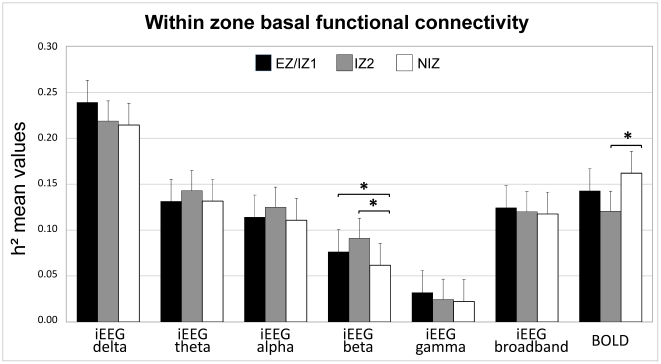
h^2^ means values within zones (within zone connectivity) for iEEG sub-bands, broadband signal and for BOLD signal. *: p<0.05, Kruskal Wallis test and Mann Whitney U test, corrected for multiple comparisons. Epileptogenic/primary irritative zone (EZ/IZ1), secondary irritative zone (IZ2) and non irritative zone (NIZ).

Functional connectivity was quantified in the form of pair-wise interactions based on the computation of non linear correlations (h^2^) on broadband and band-passed iEEG signals and on the ROI-averaged resting-state BOLD signals across subjects (see Fig. 2). h^2^ values were averaged across 15 min recordings for each modality.

### Within-zone basal functional connectivity (Fig. 2)

#### Within-zone basal functional connectivity derived from iEEG signals

We have first compared the correlations values obtained within the three considered zones (EZ/IZ1; IZ2 and NIZ).

For broadband (bb) signals, no significant differences were observed in h^2^ values, reflecting within-zone basal functional connectivity between EZ/IZ1 (mean-h^2^_bb_ = 0.124±0.080), IZ2 (mean-h^2^_bb_ = 0.120±0.080) and NIZ (mean-h^2^_bb_ = 0.118±0.070) (p>0.05, Kruskal Wallis test).

In the delta band (ð), after corrections for multiple comparisons only trends of differences in mean h^2^ values were observed with a slightly elevated functional connectivity within EZ/IZ1 (mean-h^2^_ð_ = 0.239±0.073) compared to NIZ (mean-h^2^_ð_ = 0.214±0.057) (p = 0.036 uncorrected, not surviving correction for the multiple comparisons) and to IZ2 (mean-h^2^_ð_ = 0.219±0.076) (p = 0.023 uncorrected, not surviving corrections for multiple comparison).

In the theta band (θ), no differences in mean h^2^ values were observed between EZ/IZ1 (mean-h^2^_θ_ = 0.131±0.076), IZ2 (mean-h^2^_θ_ = 0.143±0.087) and NIZ (mean-h^2^_θ_ = 0.132±0.087) (p>0.05, Kruskal Wallis test). In the same way, in the alpha band (α), no differences in mean h^2^ values were observed within EZ/IZ1 (mean-h^2^_α_ = 0.114±0.081), IZ2 (mean-h^2^_α_ = 0.125±0.075) and NIZ (mean-h^2^_α_ = 0.111±0.072) (p>0.05, Kruskal Wallis test).

In the beta band (β) relative to NIZ (mean-h^2^_β_ = 0.062±0.049), significant increases in h^2^ mean values were observed within EZ/IZ1 (mean-h^2^_β_ = 0.076±0.047) (p = 0.015, corrected for multiple comparisons) and within IZ2 (mean-h^2^_β_ = 0.091±0.061) (p = 0.005, corrected for multiple comparisons).

In the gamma band (γ), no differences in mean h^2^ values were observed within EZ/IZ1 (mean-h^2^_γ_ = 0.114±0.081), IZ2 (mean-h^2^_γ_ = 0.125±0.075) and NIZ (mean-h^2^_γ_ = 0.111±0.072) (p>0.05, Kruskal Wallis test).

#### Within-zone basal functional connectivity derived from Resting state BOLD fMRI signals

The resting state fMRI mean h^2^ value was significantly lower within IZ2 (mean-h^2^_bold_ = 0.120±0.068) compared to NIZ (mean-h^2^_bold_ = 0.162±0.068) (p = 0.005, corrected for multiple comparisons). No significant difference was observed between h^2^ values within EZ/IZ1 (mean-h^2^_bold_ = 0.143±0.073) and within NIZ (p = 0.288).

In summary iEEG-based within-zone functional connectivity appears to be elevated in EZ/IZ1 and IZ2 in the beta band relative to NIZ, whereas resting state BOLD fMRI-based within-zone functional connectivity is lower in IZ2 relative to NIZ (Fig. 2).

### Inter-zone basal functional connectivity (Fig. 3)

**Figure 3:**
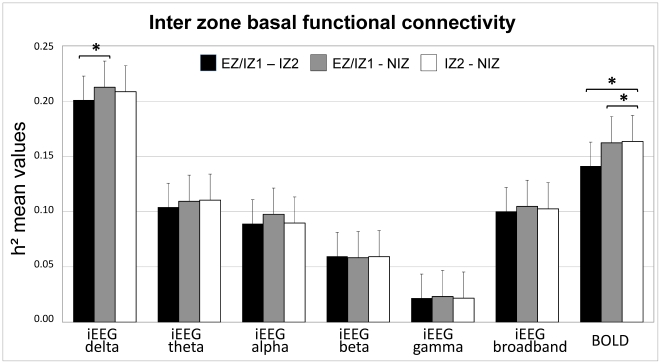
h^2^ means values between zones (inter zone connectivity) for iEEG sub-bands, iEEG broadband, and BOLD signal. h^2^ means values between (EZ/IZ1)-(IZ2), between (EZ/IZ1)-(NIZ), between (IZ2)- (NIZ). * p<0.05, Kruskal Wallis test and Mann Whitney U test, corrected for multiple comparisons.

Mean h^2^ values were determined between all pairs of ROIs belonging to two different zones relative to their epileptogenic status (EZ/IZ1, IZ2, NIZ) and averaged for each type of connections (e.g. h^2^ values for links between: i. (EZ/IZ1) and (IZ2) (degrees of freedom, dof = 98), ii. (EZ/IZ1) and (NIZ) (dof = 119), iii. (IZ2)-(NIZ) (dof = 86)) (See fig. 3).

#### Inter-zone basal functional connectivity derived from iEEG signals

For the broadband signal, no significant differences were observed in h^2^ values between the 3 zones: between (EZ1/IZ1) and (IZ2) (mean-h^2^_bb_ = 0.100±0.056), between (EZ/IZ1) and (NIZ) (mean-h^2^_bb_ = 0.105±0.052) and between (IZ2) and (NIZ) (mean-h^2^_bb_ = 0.103±0.059) (p>0.05, Kruskal Wallis test).

In the delta band, mean-h^2^_ð_ values of functional connectivity between (EZ1/IZ1) and (IZ2) (mean-h^2^_ð_ = 0.201±0.050) were significantly lower than mean-h^2^_ð_ values of connectivity between (EZ/IZ1) and (NIZ) (mean-h^2^_ð_ = 0.213±0.050) (p = 0.043, corrected for multiple comparisons), but not significantly different from mean-h^2^_ð_ values of connectivity between (IZ2) and (NIZ) (mean-h^2^_ð_ = 0.209±0.047).

No significant differences in h^2^ values were observed neither in the theta band (mean-h^2^_θ_ for connectivity between (EZ/IZ1) and (NIZ) = 0.104±0.042; mean-h^2^_θ_ for connectivity between (EZ/IZ1) and (NIZ) = 0.109±0.053; mean-h^2^_θ_ for connectivity between (IZ2) and (NIZ) = 0.110±0.047), nor in the alpha band (mean-h^2^_α_ for connectivity between (EZ/IZ1) and (NIZ) = 0.089±0.041; mean-h^2^_α_ for connectivity between (EZ/IZ1) and (NIZ) = 0.098±0.064; mean-h^2^_α_ for connectivity between (IZ2) and (NIZ) = 0.090±0.045), nor in the beta band (mean-h^2^_β_ for connectivity between (EZ/IZ1) and (NIZ) = 0.060±0.026; mean-h^2^_β_ for connectivity between (EZ/IZ1) and (NIZ) = 0.058±0.032; mean-h^2^_β_ for connectivity between (IZ2) and (NIZ) = 0.059±0.039), nor in the gamma band (mean-h^2^_γ_ for connectivity between (EZ/IZ1) and (NIZ) = 0.022±0.007; mean-h^2^_γ_ for connectivity between (EZ/IZ1) and (NIZ) = 0.023±0.008; mean-h^2^_γ_ for connectivity between (IZ2) and (NIZ) = 0.022±0.015).

#### Inter-zone basal functional connectivity derived from resting state BOLD fMRI signals

The mean h^2^ values derived from resting state BOLD fMRI were significantly decreased between (EZ1/IZ1) and (IZ2) (mean h^2^_bold_ = 0.141±0.083) relative to mean h^2^ between (EZ/IZ1) and (NIZ) (mean h^2^_bold_ = 0.162±0.080) (p = 0.023, corrected for multiple comparisons) and between (IZ2)-NIZ (mean h^2^_bold_ = 0.164±0.087) (p = 0.032, corrected for multiple comparisons).

In summary, inter-zone basal functional connectivity was found to be decreased between (EZ1/IZ1) and (IZ2) relative to inter-zone connectivity between (EZ/IZ1) and (NIZ) in both modalities (in the delta band for EEG signals and in BOLD connectivity) (Fig. 3).

### Correlations between basal functional connectivity derived from iEEG and rs-fMRI (Fig. 4)

**Figure 4:**
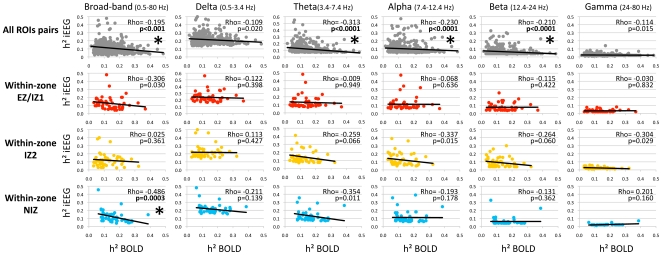
Correlations between iEEG and BOLD connectivity. Spearman rank correlations; * p<0.05, corrected for multiple comparisons.

We plotted the h^2^ values derived from iEEG against those derived from BOLD across all ROI pairs (within and inter zone connections) for each EEG band (see Fig. 4).

Pooling together all the h^2^ values characterizing the within- and the inter-zone interactions, significant negative correlations (Spearman Rank) were observed between functional connectivity parameters derived i) from BOLD and broadband iEEG signals (Rho = −0.195; p<0.001; corrected for multiple comparisons); ii) from BOLD and theta band iEEG signals (Rho = −0.313; p<0.0001, corrected for multiple comparisons) iii) from BOLD and alpha band iEEG signals (Rho = −0.230; p<0.0001, corrected for multiple comparisons) and iv) from BOLD and beta band iEEG signals (Rho = −0.210; p<0.0001, corrected for multiple comparisons). Trends for negative correlations were observed between h^2^ values derived from BOLD and delta band iEEG signals (Rho = −0.109; p = 0.02 uncorrected, not surviving correction for multiple comparisons) and BOLD and gamma band iEEG signals (Rho = −0.114; p = 0.015 uncorrected, not surviving correction for multiple comparisons).

Looking at the correlations between h^2^ values derived from BOLD and iEEG within zones, the only significant relationship was obtained within NIZ, where BOLD h^2^ values were negatively correlated with broadband iEEG h^2^ values (Rho = −0.486; p = 0.003, corrected for multiple comparisons).

### Causality between EZ1/IZ1; IZ2 and NIZ derived from iEEG and BOLD signals

To evaluate the causality between zones, we determined the mean directionality indexes (D) between all pairs of ROIs belonging to two different zones (EZ1/IZ1; IZ2 and NIZ) based on broadband iEEG and BOLD signals.

For both modalities, the mean D values were significantly different from zero from EZ/IZ to NIZ (iEEG D_mean_ = 0.062, p = 0.047; BOLD D_mean_ = 0.093, p = 0.005) reflecting a significant causality of EZ/IZ1 onto NIZ during the interictal period. No significant causality was observed for the two other interactions between EZ/IZ1 and IZ2 (iEEG D_mean_ = −0.023, p = 0.454; BOLD D_mean_ = 0.037, p = 0.254) and between IZ2 and NIZ (iEEG D_mean_ = 0.032, p = 0.297; BOLD D_mean_ = −0.035, p = 0.252).

Mean D values derived from broadband iEEG and BOLD signals were positively correlated (Spearman Rho = 0.247, p<0.0001).

**Figure 5:**
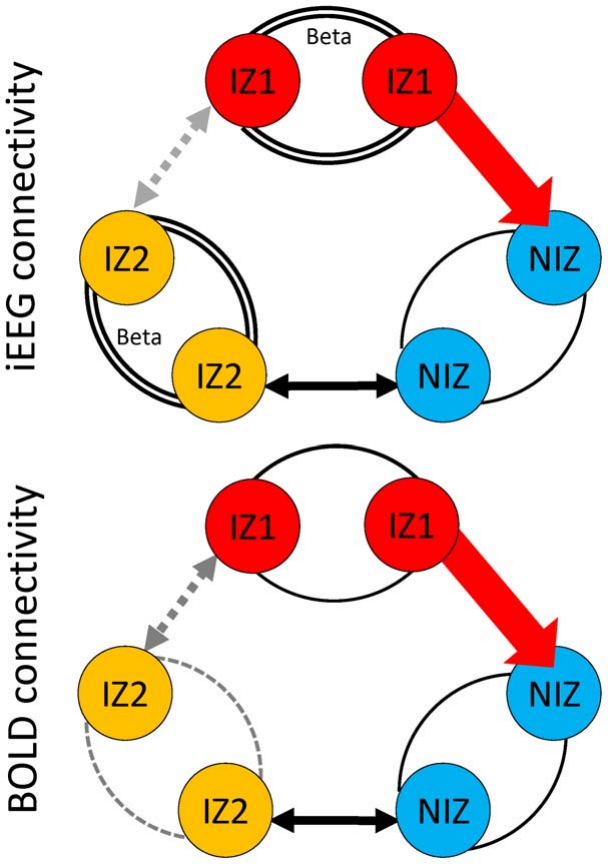
Schemes of functional (h^2^) and effective (direction index) connectivity during interictal period. Circles represent regions of interest from the different zones: EZ/IZ1: epileptogenic/primary irritative zone; IZ2: secondary irritative zone; NIZ: Non irritative zone. Within zone functional connectivity: Curved lines represent functional connectivity (h^2^ correlations) between 2 ROIs in the same zone. NIZ to NIZ connectivity is considered as ‘normal’ functional connectivity. Compared to this reference, i) double curved lines represent significantly increased functional connectivity between ROIs in other zones, ii) grey and dotted curved lines represent significantly decreased functional connectivity between ROIs in other zones, and iii) grey curved lines represent trend of decreased functional connectivity between ROIs in other zones. Inter zone functional connectivity: Arrows represent effective connectivity between zones. Unidirectional and red arrows correspond to significant direction index (effective connectivity) between zones. Double arrows correspond to non-significant direction index between zones. Grey and dotted double arrows represent significantly lower functional connectivity (h^2^ values) between IZ1-IZ2 compared to h^2^ values between other zones.

Figure 5 summarizes basal functional connectivity organization assessed by iEEG and BOLD signal and causality between zones.

## Discussion

In this study, combining results obtained from electrophysiological and fMRI techniques, we observed higher functional connectivity as estimated from iEEG and lower functional connectivity as derived from BOLD signals, between structures belonging to epileptic regions relative to regions spared by epileptiform discharges. However, both modalities showed a signal directionality suggesting an influence of the epileptic zone at rest onto remote areas actually spared by electrical abnormalities. Direct comparisons of functional connectivity derived from iEEG and BOLD signals showed significant negative correlations when considering all pairs of signals and when considering pairs of signals in regions spared by epileptiform discharges. This suggests widespread neurovascular coupling alterations in temporal lobe epilepsy.

### Issues related to inter-modality comparisons

Comparing EEG and BOLD signals is challenging and potential pitfalls must be acknowledged before discussing further the interpretation of these results. The first is the method used to study signals interdependencies. In previous studies different functional connectivity measures were used and comparisons were performed in different groups of subjects. To overcome this issue, we chose to use the same functional connectivity calculation method (h^2^) on both iEEG and BOLD data in the same patients. The use of the h^2^ method was recently evaluated with respect to those of other methods on iEEG signal [14]. One advantage of the h^2^ method, among others, is that it is applicable for either linear or nonlinear relationships between signals, in contrast to linear methods that are only sensitive to the linear component of the relationship. h^2^ can be viewed as a general method to detect and characterize potential associations between time series, whatever their origin and nature. It is therefore appropriate in situations where no *a priori* information is available about the type of relationship to be identified.

A potential limitation of this study is the fact that we have compared non-simultaneous recordings of iEEG and BOLD signals. The time between the two acquisitions ranged from 3 days to 2 months. However, we postulate that during this period of time it is unlikely that a progression of epileptogenicity has occurred, because no major events suspected to provoke short term changes in epileptogenic networks (such as status epilepticus, hypoxia or trauma) [15] were recorded during the interval. Significant progression of the epileptogenic process as shown by neuroimaging or SEEG analysis [16] is thought to take place over several years of epilepsy course.

Thus the localization of the irritative and epileptogenic zones used in the present study is likely to be identical in the two datasets. Nevertheless, the rate of interictal epileptiform discharges could be different during the two procedures and only simultaneous recording of iEEG and fMRI could address this question.

### Neural correlates of BOLD signal spontaneous fluctuations in physiological conditions

Comparison with iEEG is a powerful method to investigate the electrophysiological correlates of hemodynamic changes measured by fMRI. Concerning classical task or stimulus-related fMRI, numerous studies have demonstrated the links, though indirect, between neuronal activity and BOLD signal modulation [17], [18]. Evoked BOLD response correlates well with fluctuations in gamma power of local field potentials (LFP) [17], [19]–[22]. Conversely, there is no perfect correlation between BOLD modulation and single neuron unit firing rate [17], [23]; this kind of correlation depending on the level of local interneuronal coupling [24]. Interestingly, electrophysiological correlates of BOLD changes may differ depending on the type of cortex studied [25]. Whereas the main correlations were found in the gamma band mainly in sensory neo-cortical regions (i.e. visual, auditory and somatosensory cortices), theta band LFP power correlations were found in human hippocampus [26]. Moreover, whereas the majority of studies on the relationship between BOLD signal and underlying neuronal activity (LFP and spiking) have shown correlations, dissociations have also been reported depending on the species, the regions and the methodology used ([25] for review).

Less is known concerning the relationship between the spontaneous fluctuation of BOLD signal at rest (which is at the basis of functional connectivity MRI) and the underlying neuronal activity. There is evidence in the literature of spontaneous slow fluctuation of neuronal activity (<0.1 Hz) with a characteristic 1/f-like distribution as it is the case for the spontaneous BOLD oscillations [27]. Concordant interhemispheric correlations of neuronal spontaneous slow fluctuations have been found in human sensory cortex using iEEG. In addition, computational studies have demonstrated that <0.1 Hz fluctuation is a frequency at which the coherence of gamma oscillation is optimal [28]. To date, only one study has shown direct concurrent spontaneous fluctuations of neuronal activity and BOLD signal in Monkey visual cortex by using simultaneous fMRI and intracortical electrophysiological recording at rest [4]. Whether these data were obtained strictly at rest has been recently debated by Logothetis and colleagues that re-analyzed the same data and found “imperceptible but physiologically clearly detectable flicker induced by the visual stimulator” [29]. Therefore such positive correlations from simultaneous recordings in different types of cortex and experimental conditions remain to be confirmed. However, comparable correlations have also been obtained in humans by using non-simultaneous resting-state fMRI and electrocorticography (ECoG) recordings during the presurgical assessment of 5 patients presenting with partial epilepsies [5]. During wakefulness, the slow cortical potentials and gamma frequency power recorded by ECoG showed a correlation structure similar to that of spontaneous BOLD signal fluctuations.

Thus, as it is the case for evoked BOLD modulation, spontaneous BOLD fluctuations have been found to correlate mainly to gamma band oscillations of neuronal activity in primary cortices in animals and humans [4], [5]. However, these correlates could also differ depending on the animal species and the neural systems studied. In rats, delta band neuronal oscillations correlated to BOLD fluctuations in the sensory cortex [30]. A recent MEG study on neuronal correlates of 2 classical resting state networks defined by fMRI (i.e. attention and default mode networks), found synchronous correlation in lower frequency bands: primarily within the theta, alpha and beta bands [31].

However, in all these studies signals were extracted from cortex supposed to behave in physiological condition or at least spared by epileptic processes. Therefore, the potential influence of electrical abnormalities and the relationships between BOLD and EEG signal in epileptic cortices have never been properly addressed yet.

### Neural correlates of BOLD signal spontaneous fluctuations in temporal lobe epilepsy

Our study is therefore the first to investigate the electrophysiological basis of BOLD resting-state connectivity both in pathological and cortices spared by electrical abnormalities in humans using iEEG. Previous studies using separate populations of patients, have suggested a discrepancy between functional connectivity as measured by iEEG recording (showing an increased functional connectivity in the EZ/IZ1) [13], [32] and the one measured by resting state fMRI, showing decreased functional connectivity in the EZ/IZ1 [10]. This discrepancy is confirmed in the present study, although we have found that the increased iEEG connectivity was specific to the beta bands. The meaning of this beta synchrony increase in epileptogenic regions (in comparison with non epileptic) is unclear. Nevertheless, we postulate that this persistent increase in beta frequency observed within epileptogenic networks could be a reliable pathological marker of epileptic processes. In the literature, beta-band activity has been proposed to be involved in large-scale coordination of distributed neural activity, in particular in task requiring working memory [33], attentional processing [34] or visual conscious perception [35]. Assuming that iEEG gives a more accurate picture of neural connectivity, a possible explanation for these findings is abnormal neurovascular coupling. Indeed, the studies of Shmuel & Leopold and He et al. are performed under the assumption of normal physiological conditions, so subject to normal (canonical) neurovascular coupling [4], [5]. However, neurovascular coupling requires integrity of metabolism, vascularisation and blood-brain-barrier (BBB) that all may be affected in TLE: i. metabolic alterations have been largely demonstrated by MR spectroscopic imaging and PET [36]–[38], ii. vascularisation defects have been demonstrated by SPECT and perfusion MRI [38], iii. BBB dysfunctions have also been highlighted in TLE by the discovery of BBB transporter-protein deficit such as GLUT-1 interfering with glucose metabolism [39]. In addition, decreased structural connectivity demonstrated using morphometric correlation [40] or diffusion imaging – tractography [41]–[43] might also play a role in the decrease of functional connectivity as measured by fMRI.

We found iEEG and BOLD-derived functional connectivity to be negatively correlated: i) when considering all pairs of signals (including IZ1/EZ, IZ2 and NIZ), for broadband iEEG signals and in theta, alpha and beta sub-bands and ii) when considering only pairs of signals in regions spared by electrical abnormalities (NIZ) for broadband iEEG signals.

These observations might appear as different with those of He et al. who found a positive correlation in healthy cortices of epileptic patients. One hypothesis is that theses differences are related to the metrics (non linear versus linear) used here to quantify interrelations between pairs of signals. Indeed, it has been showed in EEG signal that different (and sometimes discrepant) results can be obtained from different methods [14]. This is why we chose the h^2^ method which does not require strong assumptions on the type of interdependence to be characterized and we applied the same metric for the different types of signal (iEEG and BOLD).

However, the more plausible explanation relative to differences with the findings of He et al. might be related not only to the type of cortex but also to the type of epilepsies studied. Indeed, He and colleagues studied motor and sensory-motor networks in patients with frontal epilepsy [5]. In our study, we included patients with TLE known to be associated to widespread with metabolic and hemodynamic alterations secondary affecting areas distant from the EZ [44]. This actually suggests that there was no real healthy cortex explored and that those spared by electrical abnormalities may also present with altered neurovascular coupling.

Our study as well as the studies of Shmuel & Leopold and He et al. actually found relatively weak correlations between functional connectivity measured by BOLD and EEG signal [4], [5]. A possible explanation is that BOLD and LFP relationships are not always linear and may change depending on factors already described above. This issue and the different methodology used to measure both functional connectivity and correlation between signals may account for the apparent discrepancies found between our studies. Correlation functions used by Shmuel & Leopold were defined based on the covariance of both signals tested with a two-tailed pair t-test (P<0.01) [4]. He et al. used a Pearson's correlation test and found a correlation between BOLD signal correlations and ECoG correlations with an r^2^ between 0.14 and 0.35 (p = 0.008 and p<0.0001) depending on the band frequency of ECoG recordings (r^2^ = 0.14, p = 0.008, for the gamma band) [5] so again a relatively weak correlation though significant. Our study also found relatively weak significant correlation using a Spearman's correlation test between BOLD and iEEG signal correlation (Rho between −0.49 and −0.20, p<0.001 or p<0.0001). The availability of simultaneously recorded iEEG-fMRI data [45] could help address the question of the effect of epilepsy on the coupling between EEG and BOLD.

The alternative but not contradictory hypothesis is that each modality actually measures different phenomena linked to epilepsy possibly occurring at different time scales. Increased functional connectivity as measured by SEEG recording in TLE might be a specific electrophysiological feature of epileptic networks not related to the functionality of such networks. Conversely, decreased functional connectivity as measured by resting-state fMRI would reflect function deficits associated to epilepsy. This would be in agreement with the decreased functional and effective connectivity in several functional networks observed in TLE patients using fMRI [6]–[12], [46].

### Effective connectivity

The functional connectivity alteration of language, perceptual, attention, default mode and memory networks that are related to, but do not perfectly overlap the EZ/IZ1, means that non epileptic areas may also be affected by dysfunctions during interictal period. Our findings concerning the direction index of EEG and BOLD signals suggesting an influence of the epileptic areas over the regions spared by epileptiform activities shed light on the underpinning mechanisms of cognitive impairment observed in patients during the interictal period.

This directionality has been estimated using the direction index derived from h^2^ measurements. This index makes use of both the asymmetry of h^2^ values and the time delay between analyzed signals. Concerning directionality, a study conducted in an animal model of absence epilepsy showed the complexity of iEEG and fMRI with regard to signal directionality that depends on the model used [47]. The advantages of the h^2^ method are twofold. First, conversely to methods based on Granger's causality, the h^2^ method is nonparametric, i.e., no model (like the classically-used multivariate autoregressive model) is required to describe underlying time series. Second, it can be applied in a pair-wise fashion and hence, is not limited to a reduced number of regions of interest as it is the case for Dynamic Causal Modelling [47]–[49].

### Effects of interictal epileptiform discharges?

Interictal spikes are event that dramatically increase the synchrony between involved structures [50]. However, in a previous study we have demonstrated an interictal increased EEG synchrony of background EEG after spikes removal [13] even if spikes were likely to play a role in the increased EEG coupling. The effect of spikes on BOLD signal is less clear. Simultaneous EEG/fMRI recordings have shown that spikes can either be associated to increased, decreased or unchanged BOLD signal [51]. The hypothesis of a neurovascular decoupling has also been questioned to explain this complexity [52]. EEG/fMRI is limited by the detection of spikes on scalp EEG and the underestimation of spikes not expressing on surface EEG. This may lead to false BOLD signal baseline. Nevertheless, it is likely that interictal spikes are at least in part responsible for the discrepancy between EEG and BOLD coupling studied here. However, the negative correlations found between both signals in regions spared by epileptiform abnormalities show that spikes are not the sole responsible.

Recent developments [45], [53] have shown the potential feasibility of simultaneous iEEG-fMRI recording and future studies using this technique would be an advantage to overcome these issues.

### Conclusion

Our study shows unexpected relationships between functional connectivity as measured from BOLD and iEEG signals in patients suffering from TLE not only in regions affected but also those spared by epileptiform electrical activities. We suggest that both techniques may be complementary and that further studies must be conducted to investigate the electrophysiological correlates of spontaneous BOLD signal fluctuation in pathological conditions.

## Methods

### Subjects

Five patients with drug-resistant temporal lobe epilepsy (3 with right TLE, 1 with left TLE, 1 with bilateral TLE) undergoing pre-surgical evaluation gave their informed consent to take part in this study approved by the local Ethics Committee.

All patients (Table 1) had a comprehensive evaluation including detailed history and neurological examination, neuropsychological testing, conventional magnetic resonance imaging (MRI), surface electroencephalography (EEG) and stereoelectroencephalography (SEEG, depth electrodes) recording as previously reported [54], [55]. In all the 5 five patients, SEEG revealed that seizures started from the mesial part of the temporal lobe and included additional cortical area in patient 1.

**Table 1:** Clinical characteristics of patients and number of regions of interest explored in each zone.

Patient #	age	gender	EZ/IZ1laterality	#Electrodes	MRI visible lesion	# of ROIs explored in each zone			
						EZ/IZ1	IZ2	NIZ	sum
1	49	M	Right	8 right	Right temporal lobe dysplasia	8	2	6	16
2	26	M	Right	8 right	No visible MRI lesion	4	3	8	15
3	39	M	Right	7 right	Right MTL cortical dysplasia	4	7	4	15
4	48	F	Bilateral	6 left2 right	Left hippocampal sclerosis	6	8	2	16
5	25	F	Left	6 left	Left hippocampal sclerosis	3	5	2	10

EZ/IZ1 = epileptogenic zone; IZ2 = secondary irritative zone; NIZ = non irritative zone.

### iEEG recordings

Intra-cerebral EEG (iEEG) recordings were performed using multiple contact depth electrodes (10–15 contacts, length: 2 mm, diameter: 0.8 mm, spaced 1.5 mm apart) positioned according to Talairach's stereotactic method [56]. Electrodes were labeled by upper case letters (A, TB, etc.) (Tab. S1), and contacts were numbered from 1 to 15 on each electrode. Low numbers (1, 2, 3, etc.) correspond to the most mesial structures (tip of the electrode, for example for electrode A, A1 and A2 correspond to contacts inside amygdala, A9 and A10 to contacts in the lateral neocortex of the middle temporal gyrus).

Electrode positions were specifically defined in each patient based upon detailed analysis of non-invasive data (surface EEG recordings, clinical symptoms, MRI) leading to hypotheses about the location of the epileptogenic zone.

iEEG signals were recorded on a 128 channels system (DeltamedTM®, France) at a sampling rate of 512 Hz. Raw signals were filtered with a high-pass hardware filter with cut-off frequency equal to 0.16 Hz at −3 dB to remove slow drifts and with a first order low-pass anti-aliasing filter (cut-off frequency equal 170 Hz at −3 dB). The resulting signal was recorded on a hard disk without any additional filtering.

The recording periods used for connectivity quantification were entirely interictal, in the awake resting state (no task) and with eyes closed (15 min recording between 09:00 am and 12:00 am). Recordings selected for analyses of interictal periods were performed at least two days after electrode implantation in order to limit possible effect of the general anesthesia, and at least 4 h distant from a seizure. Signals recorded in each patient during interictal activity were visually analyzed for each brain region of interest, and two contacts recording SEEG bipolar signal without artifact were selected. Methods for MRI data acquisition and resting state data processing are described in Data S1.

### Classification of regions according to epileptogenicity

We determined the localization of the epileptogenic/primary irritative zone EZ/IZ1, the secondary irritative zone IZ2 and the non irritative zone NIZ for each patient. The EZ/IZ1 was defined as the subset of brain sites involved in the generation of seizures and also showing interictal spiking [57]. The IZ2 zone was defined as those regions only secondarily involved in seizures and which produce interictal spikes [58]. Finally, we defined NIZ as structures without interictal spikes.

### Extraction of ROI BOLD data

**Figure 6:**
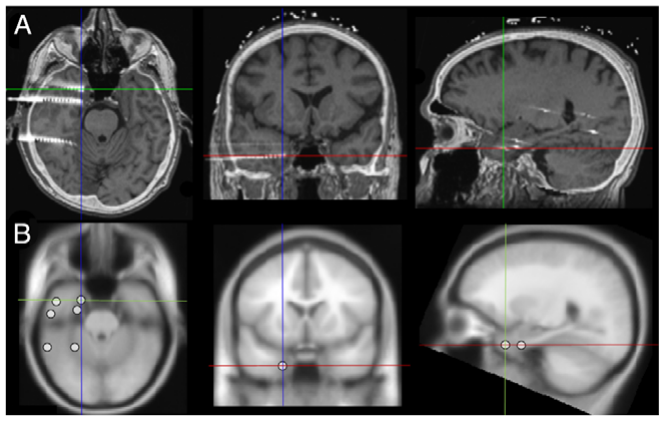
Electrodes contacts and corresponding ROI locations. (**a**) Image fusion between CT-scan obtained during implantation of electrodes and 3D T1 volume acquired after electrode implantation. (**b**) Definition of ROIs corresponding to the selected contacts.

We extracted BOLD signal from the same regions as those recorded from iEEG (Fig. 6). In summary the following procedure was used: CT scan acquired post implantation and a 3D T1-weighted volume acquired after electrodes implantation were co-registered (Medinria software). As we used a stereo-electroencephalography (SEEG) procedure, each electrode explored medial and external cortex. We selected two ROIs per electrode (fig.1): 1 medial and 1 lateral. For the iEEG signal we used the signal obtained from respectively 2 medial and 2 external electrode contacts; for BOLD signal analysis, we used a spherical ROIs with a centre located between the 2 contacts selected for iEEG signal. For each selected iEEG contact, a ROI with a radius of 5 mm centered on the contact was defined and a ROI fMRI signal time course was extracted by averaging the pre-processed fMRI over the ROI voxels (see details in Data S1).

### Estimation of nonlinear cross-correlations between iEEG signals

Functional connectivity was estimated from iEEG signals recorded from distinct structures. Using nonlinear regression analysis, we computed statistical couplings between artifact-free signals according to a pair-wise approach (i.e. on each pair of areas of interest) over the selected 15 min resting-period. Details of the method can be found in previous reports [59], [60]. In short, the dependency between two considered signals *X* and *Y* was quantified by a normalized nonlinear correlation coefficient h^2^ given by
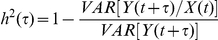
with
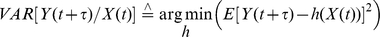
where *h* is a nonlinear fitting curve which approximates the statistical relationship between *X* and *Y* and where 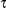 is the time shift (delay) that maximizes the value of the h^2^ coefficient. In practice, the *h* function can be obtained from the piece-wise linear approximation between the samples of the two time series. Conceptually, h^2^ quantifies the reduction of variance of signal *Y* that is obtained when *Y* samples are predicted for *X* samples. Indeed, h^2^ = 0 when there exists no relationship between *X* and *Y*, i.e. the conditional variance 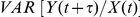 (in eq.1) is equal to the marginal variance 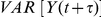. Conversely, h^2^ = 1 when signal *Y* is fully determined by signal *X*, i.e. when the conditional variance 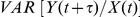 = 0 (in eq. 1).

Values were averaged over time in order to get a single estimate (mean±S.D) of the functional coupling between mesial structures. In the present study, h^2^ values were computed on both raw broadband signals (providing a global estimation of nonlinear interdependencies, named “global iEEG” in this study) and on raw signals filtered in classically defined EEG sub-bands, namely delta (0.5–3.4 Hz), theta (3.4–7.4 Hz), alpha (7.4–12.4 Hz), beta (12.4–24 Hz) and gamma (24–80 Hz). For filtered signals, Hamming finite impulse response (FIR) filters were chosen for their linear phase that is more appropriate for the computation of time delays between bandpass-filtered signals (for details see [13]).

As mentioned above, a bipolar montage was used in order to limit the influence of the common reference. Bipolar iEEG signals were obtained by subtracting iEEG monopolar signals recorded on two adjacent contacts (e.g. the bipolar signal A1–A2 derived from monopolar signals recorded at the level of contacts #1 and #2 on electrode A).

### Estimation of non-linear cross-correlations among BOLD signals

To determine functional interactions between ROIs in each patient, the same methodology (nonlinear regression) was applied. To this aim fMRI data were first resampled at a sampling frequency equal to that used in the iEEG recording (typically 256 Hz). This procedure was motivated by the use of an already-available software module to compute h^2^ values. This module (part of the AMADEUS software) was initially developed to perform h^2^ analysis on depth-EEG signals and cannot deal with sampling frequencies less than 1 Hz [14], [50], [59], [60]. From the technical point of view, the interpolation was performed using a 1-dimensional 1st order linear interpolation routine available in Matlab (interp1). In order to verify that the oversampling using linear interpolation did not affected the measured correlations we proceeded to a comparison between h^2^ values as measured on interpolated and non interpolated signals in one data set. The linear correlation between h^2^ values measured on the two pairs of bold signals (initial dataset and interpolated dataset) showed r^2^ = 0.96 (see Figure S1).

Nonlinear correlation h^2^ values were then computed over a 120 s sliding window (with a crossover of 5 s) for all possible pairs. Values were averaged over time in order to get a single estimate (mean±S.D.) of the degree of coupling between ROIs. To take into account the effect of proximity of two adjacent ROIs, and the spatial filtering applied, we corrected h^2^ values by the distance between each pair of ROIs: h^2^ values were normalized depending on the distance of each couple of ROI.

### Within and Inter-zone statistical comparisons

i.) *Within-zone comparisons*. For each modality (iEEG and BOLD) a statistical analysis of computed h^2^ values was performed in order to determine whether significant differences do exist between modalities within the above-defined zones (EZ/IZ1, IZ2, NIZ) in global iEEG h^2^ values, in frequency band-limited iEEG h^2^ values and in BOLD h^2^ values (Kruskal Wallis test, P<0.05, corrected for multiple comparisons, considering that 6 comparisons were not independent: broadband, delta, theta, alpha, beta, and gamma band iEEG signals). ii.) *Inter-zone comparisons*. We also tested differences between EZ/IZ1, IZ2 and NIZ (Kruskal Wallis test, P<0.05, corrected for multiple comparisons).

### Correlations between iEEG and BOLD connectivity

We plotted the h^2^ values derived from iEEG against those derived from BOLD across all ROI pairs, within IZ/EZ1, within EZ2 and within NIZ for each EEG band (Spearman's correlation).

### Direction indices between EZ/IZ1, IZ2 and NI areas

Directionality of functional coupling may be estimated through the nonlinear regression method [60]. To this aim, a quantity, referred to as the direction index D, takes into account both the estimated time delay between signals X and Y (latency) and the asymmetrical nature of the nonlinear correlation coefficient h^2^ (values of the h^2^ coefficient are different when the computation is performed from signal X to signal Y versus Y to X). Values of parameter D range from −1.0 (X is driven by Y) to 1.0 (Y is driven by X).

Direction indices were determined for significant couplings between (EZ/IZ1), (IZ2) and (NIZ). The significance threshold for h^2^ was defined on the linear part of the h^2^ formula such as:
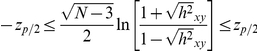
where z is the normalized normal variable, N is the total number of interactions and h^2^_xy_, the h^2^ value between pairs of ROIs. It follows that values of z outside the interval [−2.576, +2.576] constitute evidence for the existence of a correlation between variables of interest at the 99% level of confidence (p-value = 0.01, z 0.005 = −2.576). This statistical test was applied to both iEEG and BOLD h^2^ values.

The thresholding procedure did not affect the global results of brain connectivity within EZ/IZ1, IZ2 and NIZ areas described in the previous section as shown in the supplementary data. Direction indices associated with inter-regional connections were computed on both iEEG and BOLD signals. They were averaged over time in order to get a single estimate (mean±S.D.) for each modality over the respective recording sessions.

Mean direction indices were tested according to the null hypothesis in order to determine for each modality (iEEG or BOLD) whether causal relationships were present in resting-state interictal periods between EZ/IZ1, IZ2 and NIZ. Pairwise correlations were finally calculated to determine possible intermodal relationships between direction indexes obtained using BOLD and iEEG signals (Spearman Rho, P<0.05).

## Supporting Information

Data S1 Supplementary methods for conventional MRI; Resting state functional connectivity MRI (fcMRI) data acquisition and processing; and Step by step methodology to extract MRI data from regions equivalent to those explored by iEEG.
(DOC)

Figure S1 Comparison between h^2^ computed on non-interpolated and interpolated BOLD signals in one dataset. A and B show BOLD signals extracted from 2 different ROIs. C shows the similarity of the h2 values computed between signals from ROI1 and ROI2 on interpolated and non-interpolated data. D shows the linear correlation (r^2^) between the h^2^ computed on non-interpolated and interpolated data.
(TIFF)

Table S1 Detail of ROIs recorded on selected electrode contacts for each patient. Initials correspond to medial areas recorded by the electrode. Electrodes contacts numbers correspond to bipolar signal selected based onto visual analyze and representing the most representative of iEEG cortical activity and with the fewer artifacts. In patient 4, ‘ corresponds to electrodes on the right hemisphere.
(DOC)
