# Supplementary material for: Interictal Functional Connectivity of Human Epileptic Networks Assessed by Intracerebral EEG and BOLD Signal Fluctuations
Source: PLoS One. 2011 May 19;6(5):e20071. doi: 10.1371/journal.pone.0020071 (PMC3098283; doi:10.1371/journal.pone.0020071)
Supplement: Data S1 — Supplementary methods for conventional MRI; Resting state functional connectivity MRI (fcMRI) data acquisition and processing; and Step by step methodology to extract MRI data from regions equivalent to those explored by iEEG. (DOC) [file pone.0020071.s001.doc]

**Supporting information**

Bettus et al.

**Interictal functional connectivity of human epileptic networks assessed by intracerebral EEG and BOLD signal fluctuations.**

SI Text

**Supplementary methods. *Conventional MRI*.** Before SEEG exploration, patients underwent a MRI examination (duration: 80 min) as part of a multimodal MRI protocol on a 1.5T Magnetom Vision MR-scanner (Siemens, Erlangen Germany). Conventional MRI included T1-weighted images (TE/TR = 15ms/700 ms, 23 contiguous slices, 5mm slice thickness, field of view (FOV) 240 mm, matrix 256) acquired in the AC-PC plane, T2-weighted images (TE/TR = 112/7308 ms, FOV 240 mm, matrix 256, 23 contiguous slices, 5 mm slice thickness) acquired in the bihippocampal plane, T1-weighted inversion recovery images (TE/TR = 60/8000 ms, TI = 350 ms, FOV = 240mm, matrix 512, 5mm slice thickness), FLAIR images (TE/TR = 110/8000 ms, TI = 2500 ms, FOV = 240 mm, matrix 256, 5 mm slice thickness) acquired in a coronal axis perpendicular to the bihippocampal plane, and sagittal 3D-MPRAGE images (TE/TR = 4/9.7 ms, isotropic voxel of 1.25x1.25x1.25 mm3).

***Resting state functional connectivity MRI (fcMRI).*** Data acquisition and processing: Two hundred brain volumes were acquired using a single-shot multislice gradient-echo echo-planar imaging (GE-EPI) sequence (TE 55ms, TR 4s, 30 contiguous slices, 4mm-thickness, matrix 64, FOV 256mm, time elapsed between blocks: 4s). Subjects were instructed to simply keep their eyes closed and to not fall asleep during the 13 minute acquisition procedure. Data processing: Resting-state fcMRI acquisitions were pre-processed using SPM2 software (Wellcome Trust Center, London, UK). After slice timing correction, images were realigned before spatial normalization (16 non linear registration 7x6x7 basis functions) and smoothing (12 mm). Sources of spurious or regionally non-specific variance related to physiological artefacts (for example, CSF pulsations or head movement) were removed by regression including the signal averaged over the lateral ventricles and the signal averaged over a region centered on the deep cerebral white matter, to reduce non-neuronal contributions to BOLD correlations (17, 40, 41).

***Step by step methodology to extract MRI data from regions equivalent to those explored by iEEG.***

1-Realign CT scan and post-MRI: For each patient, ROIs were defined depending on the localization of depth electrodes contacts from which iEEG signals were recorded. A per-iEEG computerized tomography (CT) scan was used to check the location of each contact. After removal of depth electrodes, a MRI (post-MRI) was performed, permitting visualization of the trajectory of each electrode. In order to realign CT-scan image onto post-MRI image, we used manual landmarks. Landmarks were (i) anatomical, i.e. salient and accurately locatable points of the morphology of the visible anatomy (ii) based on CT scan depth electrodes implantation onto the skull, still visible on the post-MRI data after removal of depth electrodes. Finally, a rigid transformation was applied to the CT scan image to realign it exactly onto the post-MRI image, using MEDINRIA software (http://www-sop.inria.fr/asclepios/software/MedINRIA/).

2-Create ROIs on post-MRI: First, each contact along the electrode trajectory was localized on the CT scan image, and we created spherical ROIs centered between the two electrode contacts of each analyzed bipolar iEEG signal (MRICRO software). Secondly, ROIs were transferred onto post-MRI and regions with ROIs were delated. The resulting image was a post-MRI with ROIs marks, corresponding to black holes, on which we were able to find future ROIs location after the following post-processing:

3-Project ROIs onto resting state fMRI images: Post-MRI with ROIs were coregistered with resting-state fMRI (after timing correction and realignement). Then, we normalized this coregistered post-MRI with ROIs marks with template and resting-state fMRI. On the resulting image, we visually detected ROI localizations and created spherical ROIs (sphere radius =5mm) centered on these marks.
