# Supplementary figures and images for: Interictal Functional Connectivity of Human Epileptic Networks Assessed by Intracerebral EEG and BOLD Signal Fluctuations
Source: PLoS One. 2011 May 19;6(5):e20071. doi: 10.1371/journal.pone.0020071 (PMC3098283; doi:10.1371/journal.pone.0020071)

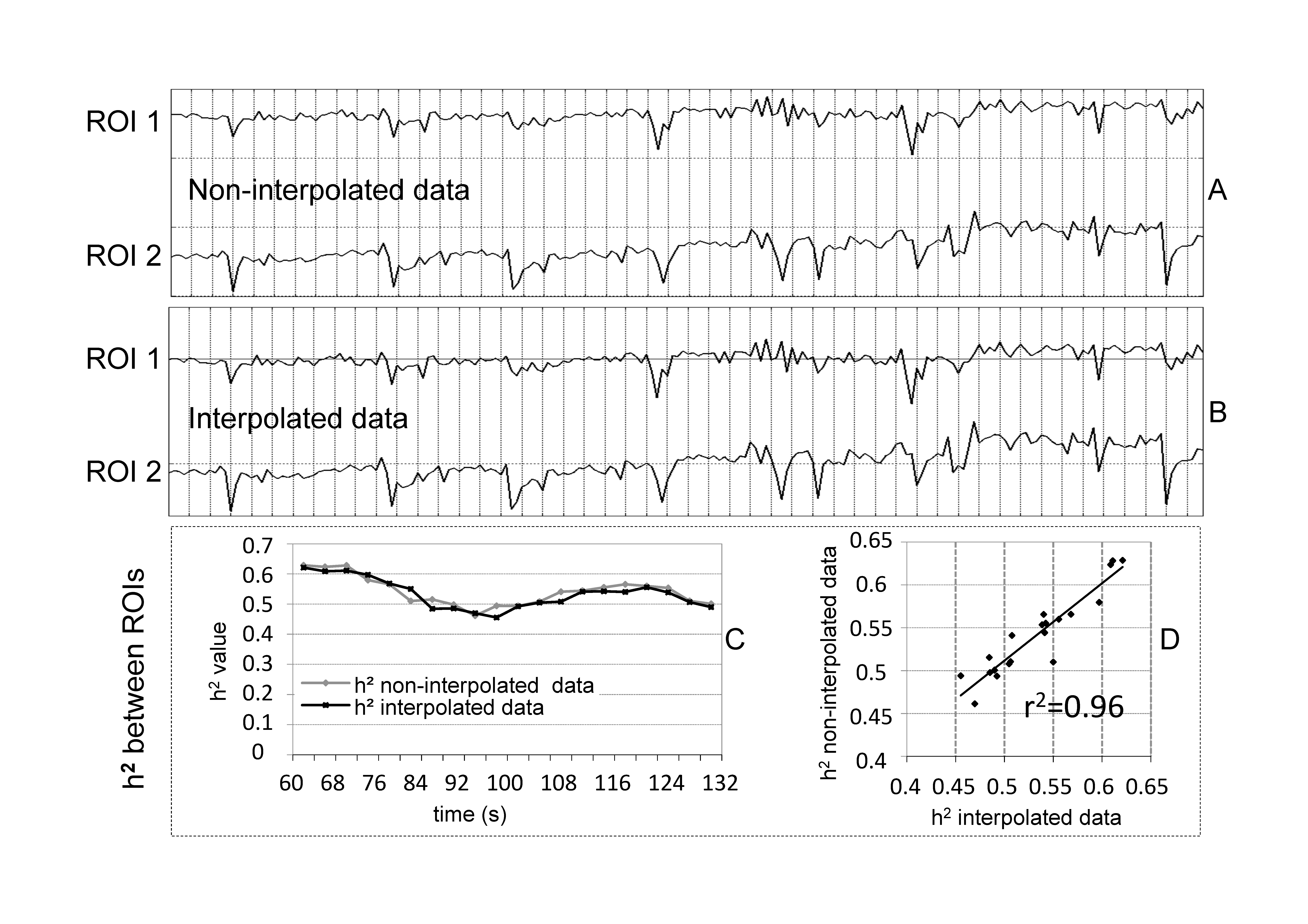

Supplement: Figure S1 — Comparison between h2 computed on non-interpolated and interpolated BOLD signals in one dataset. A and B show BOLD signals extracted from 2 different ROIs. C shows the similarity of the h2 values computed between signals from ROI1 and ROI2 on interpolated and non-interpolated data. D shows the linear correlation (r2) between the h2 computed on non-interpolated and interpolated data. (TIFF) [file pone.0020071.s002.tif]
