# Supplementary material for: Interictal Functional Connectivity of Human Epileptic Networks Assessed by Intracerebral EEG and BOLD Signal Fluctuations
Source: PLoS One. 2011 May 19;6(5):e20071. doi: 10.1371/journal.pone.0020071 (PMC3098283; doi:10.1371/journal.pone.0020071)
Supplement: Table S1 — Detail of ROIs recorded on selected electrode contacts for each patient. Initials correspond to medial areas recorded by the electrode. Electrodes contacts numbers correspond to bipolar signal selected based onto visual analyze and representing the most representative of iEEG cortical activity and with the fewer artifacts. In patient 4, ‘ corresponds to electrodes on the right hemisphere. (DOC) [file pone.0020071.s003.doc]

| Patient # 1 | | | | |
| --- | --- | --- | --- | --- |
| Electrodes | Medial contacts | Explored area | Lateral contacts | Explored area |
| TP | 2-3 | Temporal pole | 9-10 | Temporal pole |
| A | 2-3 | Amygdala | 9-10 | Middle temporal gyrus |
| TBA | 1-2 | Entorhinal cortex | 10-11 | Inferior temporal gyrus |
| B | 1-2 | Anterior hippocampus | 10-11 | Middle temporal gyrus |
| T | 2-3 | Thalamus | 11-12 | Superior temporal gyrus |
| TBP | 5-6 | Fusiform gyrus | 9-10 | Middle temporal gyrus |
| GL | 1-2 | Lingual gyrus | 8-9 | Parietal inferior lobule |
| CG | 1-2 | Posterior cingulate gyrus | 7-8 | Parietal inferior lobule |

| Patient # 2 | | | | |
| --- | --- | --- | --- | --- |
| Electrodes | Medial contacts | Explored area | Lateral contacts | Explored area |
| B | 1-2 | Anterior hippocampus | 11-12 | BA 20 |
| H | 2-3 | Heschl’s gyrus | 10-11 | Superior temporal gyrus |
| Op | 1-2 | Insular cortex | 6-7 | Parietal operculum |
| PA | 1-2 | Precuneus | - | - |
| PFG | 2-3 | Posterior cingulate gyrus | 9-10 | Parietal inferior lobule |
| TBA | 1-2 | Entorhinal cortex | 9_8 | Inferior temporal gyrus |
| TBP | 2-3 | Fusiform gyrus | 11-12 | Middle temporal gyrus |
| TP | 2-3 | Temporal pole | 9-10 | Temporal pole |

| Patient # 3 | | | | |
| --- | --- | --- | --- | --- |
| Electrodes | Medial contacts | Explored area | Lateral contacts | Explored area |
| B | 2-3 | Anterior hippocampus | 10-11 | Middle temporal gyrus |
| TBA | 2-3 | Entorhinal cortex | 10-11 | Inferior temporal gyrus |
| TBP | 1-2 | Fusiform gyrus | 7-8 | Middle temporal gyrus |
| TBP middle | 4-5 | Fusiform gyrus | - | - |
| T | 1-2 | Thalamus | 7-8 | Superior temporal gyrus |
| FCA | 1-2 | Anterior calcarine fissure | 9-10 | Occipital lateral lobe |
| OP | 3-4 | Insula | 9-10 | Parietal operculum |
| PA | 1-2 | Precuneus | 7-8 | Posterior cingulate area |

| Patient # 4 | | | | |
| --- | --- | --- | --- | --- |
| Electrodes | Medial contacts | Explored area | Lateral contacts | Explored area |
| TP | 1-2 | Temporal pole | 8-9 | Temporal pole |
| A | 1-2 | Amygdala | 11-12 | Middle temporal gyrus |
| B | 2-3 | Anterior hippocampus | 9-10 | Middle temporal gyrus |
| C | 2-3 | Posterior hippocampus | 9-10 | Middle temporal gyrus |
| TBA | 1-2 | Entorhinal cortex | 10-11 | Inferior temporal gyrus |
| T | 1-2 | Thalamus | 9-10 | Superior temporal gyrus |
| TBA’ | 1-2 | Entorhinal cortex | 9-10 | Inferior temporal gyrus |
| C’ | 1-2 | Posterior hippocampus | 7-8 | Middle temporal gyrus |

| Patient # 5 | | | | |
| --- | --- | --- | --- | --- |
| Electrodes | Medial contacts | Explored area | Lateral contacts | Explored area |
| A | 1-2 | Amygdala | 10-11 | BA 21 |
| TBA | 1-2 | Entorhinal cortex | 8-9 | Inferior temporal gyrus |
| B | 1-2 | Anterior hippocampus | 9-10 | Middle temporal gyrus |
| C | 2-3 | Posterior hippocampus | - | - |
| TBP | 1-2 | Fusiform gyrus | - | - |
| GC | 2-3 | Posterior cingulate gyrus | 10-11 | Inferior parietal lobule |

**Tab. S1.** Detail of ROIs recorded on selected electrode contacts for each patient. Initials correspond to medial areas recorded by the electrode. Electrodes contacts numbers correspond to bipolar signal selected based onto visual analyze and representing the most representative of iEEG cortical activity and with the fewer artifacts. In patient 4, ‘ corresponds to electrodes on the right hemisphere.
